# Using a Novel Approach to Estimate Packing Density and Related Electrical Resistance in Multiwall Carbon Nanotube Networks

**DOI:** 10.3390/nano10122350

**Published:** 2020-11-26

**Authors:** Usha Philipose, Yan Jiang, Gavin Farmer, Chris Howard, Michael Harcrow, Chris Littler, Vincent Lopes, Athanasios J. Syllaios, Ashok Sood, John W. Zeller

**Affiliations:** 1 Department of Physics, University of North Texas, Denton, TX 75077, USA; yanjiang@my.unt.edu (Y.J.); gavinfarmer@my.unt.edu (G.F.); ChristopherHoward7@my.unt.edu (C.H.); michael.harcrow@unt.edu (M.H.); chris.littler@unt.edu (C.L.); Vincent.lopes@unt.edu (V.L.); athanasios.syllaios@unt.edu (A.J.S.); 2 Magnolia Optical Technologies, Inc., Woburn, MA 01801, USA; aksood@magnoliaoptical.com (A.S.); jwzeller@magnoliaoptical.com (J.W.Z.)

**Keywords:** carbon nanotubes, multi-walled, electron transport, percolation limits, tunneling, fractal dimension, packing density

## Abstract

In this work, we use contrast image processing to estimate the concentration of multi-wall carbon nanotubes (MWCNT) in a given network. The fractal dimension factor (D) of the CNT network that provides an estimate of its geometrical complexity, is determined and correlated to network resistance. Six fabricated devices with different CNT concentrations exhibit D factors ranging from 1.82 to 1.98. The lower D-factor was associated with the highly complex network with a large number of CNTs in it. The less complex network, having the lower density of CNTs had the highest D factor of approximately 2, which is the characteristic value for a two-dimensional network. The electrical resistance of the thin MWCNT network was found to scale with the areal mass density of MWCNTs by a power law, with a percolation exponent of 1.42 and a percolation threshold of 0.12 μg/cm${}^{2}$. The sheet resistance of the films with a high concentration of MWCNTs was about six orders of magnitude lower than that of less dense networks; an effect attributed to an increase in the number of CNT–CNT contacts, enabling more efficient electron transfer. The dependence of the resistance on the areal density of CNTs in the network and on CNT network complexity was analyzed to validate a two-dimension percolation behavior.

“The views, opinions and/or findings expressed are those of the author and should not be interpreted as representing the official views or policies of the Department of Defense or the U.S. Government.”

## 1. Introduction

Following the discovery of carbon nanotubes (CNTs) in the mid-1970s [1], the work of Iijima in 1991 [2] triggered global attention and widespread research on CNTs. In 2013, researchers from Stanford University demonstrated a simple, functional computer made entirely from CNT-based transistors [3]. In addition to their electronic properties, the unique opto-electronic properties of CNTs have also been exploited for the development of nanoscale photovoltaic materials [4,5], photodetectors [6,7], CNT-based bolometers [8,9,10] and composite (CNT-polymer) bolometers [11,12,13]. From an application point of view, the most promising trend is using CNTs in the design of bolometer elements for infrared imaging. This is attributed to the excellent bolometric characteristics of CNTs and CNT-based composite materials as demonstrated in several studies [14,15,16]. There are several reports on CNT-polymer composites, where the optical/electrical properties of high aspect ratio CNTs, as well as the versatility of polymeric materials are exploited to significantly enhance the device potential of the composite [17,18,19,20]. The electrical conductivity of such composites is reported to be comparable to that of semiconductors [18,19,20], and in some cases attain values of several hundreds of S/m [21,22,23,24,25,26]. As the CNT concentration in the composite increases, its electrical conductivity increases sharply from nearly zero to several orders of magnitude. This increase is attributed to the network reaching a percolation threshold value due to the increased connectivity between the CNTs [27,28,29,30]. An important factor to be considered in making such composites is the dispersion of CNTs in the polymer host. Gojny et al. [31] have shown that MWCNTs have higher volume fraction and better dispersal quality compared to SWCNTs, increasing their device merit. Although devices made of single strands of CNTs would be superior in some aspects, they are not commercially viable due to lack of device reproducibility stemming from variations in chirality and geometry. Such challenges are removed in CNT networks, where the structural variations in individual CNTs are suppressed by the ensemble, as the properties are averaged over a large number of CNTs. The composites can be mass produced at low cost and high efficiency with reproducible results. A second factor that merits consideration is the curled nature (waviness) of MWCNTs. When CNTs are curled up in a matrix, they tend to have more contact points with each other. Using Monte Carlo simulations, Li et al. [25] showed that the electrical conductivity of composites with wavy nanotubes are lower than that of composites with straight nanotubes. In composite systems with dispersed CNTs, the total resistance of the film depends on the resistance of the CNT, as well as on CNT–CNT contact resistance. In such composites, a thin insulating polymer film will likely coat the CNT surfaces. If this film is thin enough, it will allow electrons to tunnel through it, but it would increase the CNT–CNT contact resistance. A study on the effect of tunneling resistance on electrical conductivity of CNT-based composites sets the upper limit of the insulating film thickness for electric tunneling as 1.8 nm [32]. The role of the polymer in determination of percolation threshold and overall film conductivity is not very clear.

Here, we investigate a MWCNT network that is not embedded in a polymer matrix, but is deposited/distributed on an SiO${}_{2}$ or quartz surface. This is the basis for many devices, including bolometer applications where CNTs are dispersed on an insulating substrate. The CNT strands are held together by Van der Waals forces; this configuration allows for a study of the electrical properties of CNT networks without the complexity of a polymer shell around the CNTs. Since MWCNTs are usually hydrophobic, they cannot be easily dispersed in water, and are treated with a surfactant to keep them homogeneously dispersed. In order to improve CNT–CNT contact resistance, the surfactant is removed and the electrical properties of the CNT network are analyzed to verify two-dimensional percolation behavior.

A mechanical dispersion technique of meniscus dragging deposition (MDD) [33] was used to distribute MWCNTs over a given substrate and fabricate devices with different packing density. We also present a strategy to quantify the dispersed CNTs and use the percolation theory to explain the electrical behavior of the MWCNT network as consisting of a number of CNTs randomly oriented with respect to each other and, in some cases, within tunneling distance of each other. An insulator-to-conductor transition was observed as the packing density of MWCNTs within the network was gradually increased. For a direct measurement of the percolation threshold, it is important to develop a technique to determine the concentration of tubes on the substrate surface. Normally the volume fraction of CNTs is determined directly by manually counting them [34], or indirectly by optical absorption measurements [35], or by numerical estimate based on mathematical modeling [36]. In this work, we present a novel strategy of using contrast imaging (black/white) for a quantitative estimate of CNTs in a given network. This technique of image analysis uses pixels of colors to detect objects of consistent color values. Finally, the fractal dimensions of the CNT networks is calculated using conventional box-counting method [37] and correlated to network resistance. The fabricated devices are comprised of only MWCNTs (without the presence of any insulating film) and the measured resistance is experimentally verified to originate from individual CNTs and from CNT–CNT contacts.

## 2. Materials and Methods

The CNTs used in this work were surfactant-treated, thin semiconducting MWCNTs (batch No. MW35-056) purchased from Nano-Integris. The CNTs were treated with the surfactant sodium dodecyl-sulfate (SDS) to ensure uniform homogeneous dispersion of the MWCNTs in water. The diameter of each MWCNT strand was about 30 nm, which includes the thickness of the surfactant shell. The concentration of the ”parent” solution was reported as 0.1 g/100 mL, which was subsequently diluted to 10 % of its original concentration. All dilution and dispersion experiments were carried out by mixing appropriate amounts of the parent suspension with de-ionized water.

**Figure 1:**
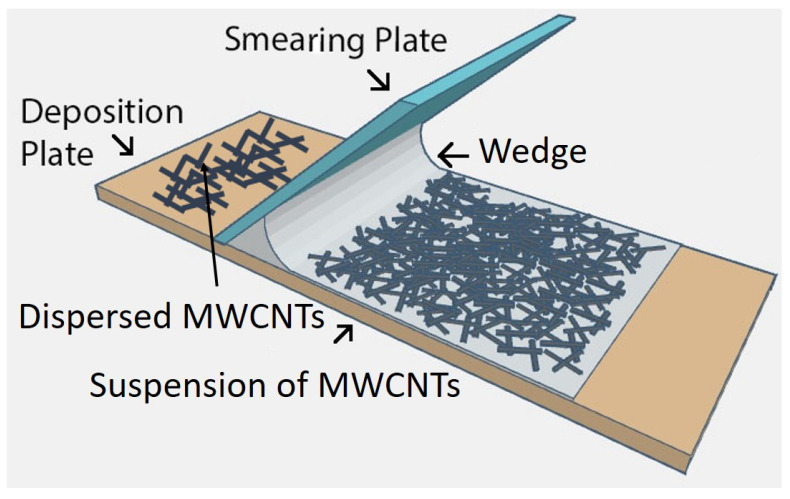
Schematic illustration of the meniscus-dragging deposition (MDD) method for fabricating MWCNT networks [33].

The as-purchased CNTs are held together in bundles or entanglements comprising of tens to a few hundred individual CNTs. These bundles would result in poor electrical properties in comparison to theoretical estimates of values related to individual CNTs. The first challenge was to disentangle the CNTs and assemble them as a network of varying packing densities on an Si/SiO${}_{2}$ or quartz substrate, without compromising their size. To separate individual CNTs from CNT agglomerates’ the parent suspension was sonicated for approximately 2 min in an ultrasonic bath (Branson 1510), running at about 42 kHz with a power of 70 W. This caused CNTs located at the outer sections of the agglomerates to be ”peeled apart”, resulting in some disentanglement of CNT agglomerates. To fabricate a large area network or film of MWCNTs, a small amount of the suspension was placed at the edge of an Si/SiO${}_{2}$ substrate and a meniscus-dragging deposition (MDD) technique was applied. This relatively simple technique can easily be adapted for mass-scale dispersion of CNTs. The technique results in randomly oriented CNTs that spread over the entire surface of the substrate. Two substrates referred to as the smearing and deposition plates were used’ such that the smearing substrate was placed on the deposition substrate at an angle of about 30${}^{\circ }$. The CNT solution was injected into the wedge between the two plates. As the smearing plate was dragged over the deposition substrate, the meniscus of the trapped suspension fluid was smeared over the deposition substrate and a thin layer of the MWCNT solution was covered it. By varying the CNT concentration in the suspension, it was possible to control the packing density of CNTs deposited on the substrate. A schematic of the MDD set up is shown in Figure 1.

Following the dispersion of the CNTs onto a chosen substrate, the physical characteristics of the CNT network was investigated using scanning electron microscope (Hitachi SU 1510 SEM operating at 10 kV). The SEM images were then converted to a binary image (black and white) using Photo-Shop (PS) Color Contrast software. This contrast imaging technique of color thresholding allowed the creation of a binary image from the SEM image, and allowed us to separate pixels containing CNTs from the background pixels. This binary image was subsequently analyzed by Image-J software [38,39]. The ‘’color count” module in Image-J calculated the pixel number for each ‘’color” and provided a quantitative assessment of the percentage coverage of CNTs in the network. Following image processing, the Fractalyse software (http://www.fractalyse.org/) was used to correlate the areal density of CNTs in the network to the fractal dimension of the CNT network using a conventional box-counting method. The fractal dimension number that represents a measure of the CNT network complexity (packing density) [37] was studied as a function of the network resistance to verify 2D percolation behavior.

To study the electrical properties of the CNT network, contact pad patterns for electrical leads were generated by photo-lithography on the Si/SiO${}_{2}$ or quartz substrate that had different packing density of CNTs on their surface. About 100 nm of gold (Au) was deposited on the contact area by thermal evaporation. The two electrical contacts were about 2 μm apart. Electrical measurements to study the properties of the fabricated device were made using an Agilent B1500 semiconductor parametric analyzer.

## 3. Results and Discussion

This section includes results from the dispersion technique as well as an analysis of electron transport through the MWCNT network along with strategies to quantify the CNTs in the network.

### 3.1. MWCNT Dispersion and Effect of Surface Treatment for Removal of Surfactant Shell

About 5.0 μL of the MWCNT solution was injected by a syringe between the deposition substrate (hydrophilized by plasma treatment) and the smearing plate. The micro-liter droplet was trapped by the capillary force and, as the smearing plate was moved back and forth across the deposition substrate, the meniscus of the trapped droplet was dragged across it, causing a thin layer of MWCNTs to coat the substrate. Since the CNTs in the parent suspension were treated with SDS (surfactant), its removal is a significant step prior to analyzing electrical transport through individual CNT strands comprising the network. For surfactant removal, the samples were treated alternately with acetone [40] and hot water three times, each treatment lasting for 15 min. The amount of acetone/water rinsing was determined by visual indication—when no more bubbles of the surfactant were observed from the network. The substrate with the dispersed CNTs was then heated at 100 ${}^{\circ }$C for 30 min to ensure that the CNT network adhered well to the substrate and to remove any residual SDS and moisture from CNT surface. Another method for SDS removal from the CNT surface is treatment with concentrated nitric acid [41,42]. However, this acidifies the surfactant salt and creates a by-product that requires removal. Rossi et al. [40] have reviewed various SDS removal techniques and summarized the effect of reagents on SDS:SWCNT interaction in solution.

**Figure 2:**
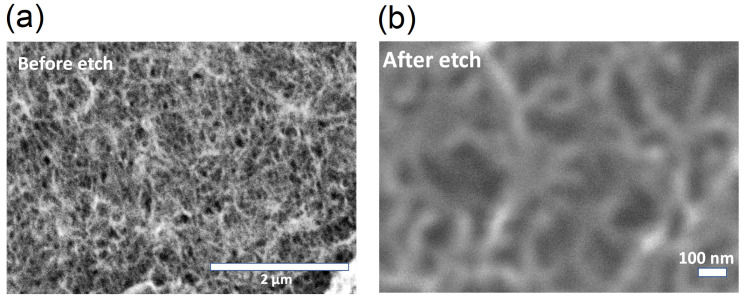
SEM images of MWCNT networks deposited on Si/SiO${}_{2}$ substrate: (**a**) before surfactant removal. The CNTs have diameters over 60 nm and the bundles are CNT agglomerates; (**b**) after surfactant removal. The diameter of the CNTs is reduced to about 30 nm and they appear to have relatively smooth surfaces.

SEM images of the MWCNT networks on the Si/SiO${}_{2}$ substrate following surfactant removal treatment are shown in Figure 2.

**Figure 3:**
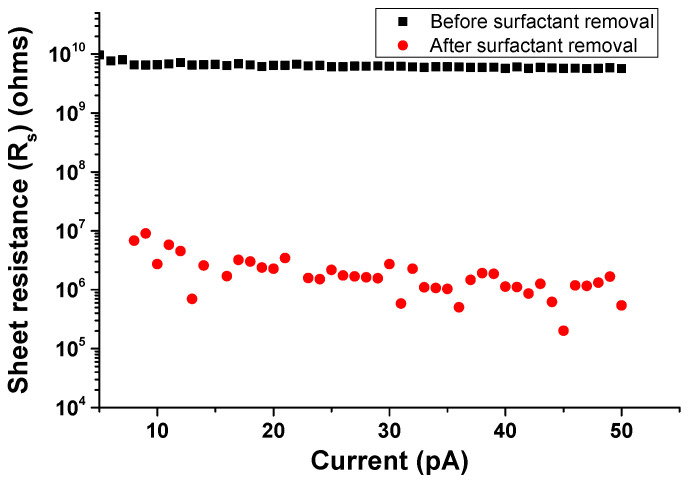
Sheet resistance of the CNT film measured before and after surfactant removal treatment. $R_{s}$ decreased about four orders of magnitude after treatment.

As seen in the SEM image, following surfactant removal, the CNTs in the network have relatively smooth surfaces. Their lengths range from 1.0 to 1.5 μm and their diameters are about 30 nm. To verify the efficacy of the surfactant removal technique, a dense network of CNTs was dispersed onto a glass slide and four contact pads were established to contact the CNT network. The glass slide was used to ensure that the network was on a highly insulating substrate. The sheet resistance of the films before and after the treatment was characterized by Van der Pauw technique using the four source monitoring units (SMUs) on an Agilent B1500 system. As seen in Figure 3, the sheet resistance of the MWCNT network before surfactant removal was of the order of $10^{10}$Ω. Following surfactant removal treatment, this value decreased to the order of $10^{6}$Ω. This result proves that treating surfactant-coated CNTs with acetone and hot water is effective in the removal of the surfactant and results in improving the electrical conductivity of the CNT network by reducing the contact resistance at the CNT–CNT junctions.

### 3.2. Percolation Limits and Electrical Resistance of MWCNT Networks with Different Packing Densities

**Figure 4:**
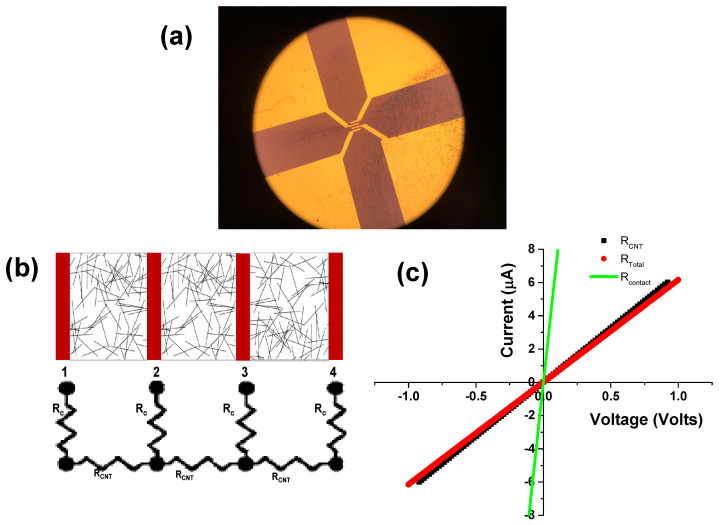
(**a**) Optical image of the four metal contacts deposited on top of the MWCNT network, (**b**) Schematic showing the series combination of CNTs and contacts represented as resistors, (**c**) Current-voltage characteristics for the MWCNTs, and the series combination of the metal contacts and CNTs. The difference between the total resistance and the CNT resistance provides the contact resistance.

Prior to measuring the resistance of the CNT network, it was important to verify that the measured resistances were due to the CNTs in the network and the contact resistance at the Au/CNT interface was negligible in comparison. For this, the MWCNTs were dispersed on a quartz substrate and a four probe configuration [43] (Kelvin probe) was used to determine the contact resistance. An optical image of the four probe contacts with a uniform distribution of CNTs in the channel is shown in Figure 4a. Due to low resolution of the optical microscope, the CNTs are not visible in the channel. A schematic of the measurement platform is shown in Figure 4b, where the CNT network resistance was determined by the equation, $R_{CNT}$ = $V_{23}/I_{14}$, is about 0.17 MΩ. In this equation $V_{23}$ is the voltage measured between terminals 2 and 3 as a current $I_{14}$ flows between terminals 1 and 4. Next, we consider the current-voltage of the series combination of the CNT network and the two end-metal contacts by applying a potential $V_{23}$ across terminals 2 and 3 and by measuring the current $I_{23}$ through the series network. Terminals 1 and 4 are kept floating during this measurement. The total series resistance of the CNT and contacts is $V_{23}/I_{23}$ = 2($R_{C}$) + $R_{CNT}$, where $R_{C}$ is the contact resistance between metal-CNTs at pads 2 and 3. The total resistance is estimated to be about 0.18 MΩ. The current–voltage characteristics determining the total resistance and the CNT network resistance are shown in Figure 4c. The difference between the total and the CNT network resistance gives a measure of the contact resistance, which is estimated to be 5 kΩ, about 36 times lower than the measured total resistance.

The total measured resistance is therefore dominated by the CNT network resistance, which includes resistance of individual CNT and CNT–CNT junctions in the network. The contribution from the metal contacts to CNTs is much lower and for all practical purposes can be neglected. Annealing the sample in air at 130 ${}^{\circ }$C for 15 min decreased the CNT–CNT contact resistance by 50 kΩ, but the metal-CNT contact resistance was relatively unaffected. It should be noted that in all the fabricated devices for measurement of electrical resistance the network comprised of a monolayer of interconnected MWCNTs. Hence the thickness of these films is estimated to be the diameter of the MWCNTs—approximately 30 nm.

To explore the effect of MWCNT packing density on the CNT network resistance, and to experimentally validate percolation limits, six devices were fabricated by dispersing MWCNTs onto a Si/SiO${}_{2}$ substrate by the MDD technique. The CNTs are randomly oriented with no preferential directions. This allows for the application of the percolation theory of a random distribution of conducting rods to the CNT network. The only requirement for this theory to be applied is that the length of the conducting rods must be greater than their diameter. The average length of the CNTs in the network is about 1.5 μm and its diameter is about 30 nm, yielding an aspect ratio of about 50. The SDS surfactant on the CNT surface was removed by the acetone and boiling water treatment, described in the preceding section. Each device was comprised of a network of CNTs contacted by two Au electrodes. The electrodes were about 3.0 μm long and 2.0 μm apart. The DC resistance (R) between the two gold contacts was measured by a standard two-probe measurement. The MWCNTs used in this study are not long enough to cross the 2 μm channel and contact the two electrical contact pads. Therefore, a network of MWCNTs distributed randomly across the channel could only carry the current measured across the channels. It was verified that the contact resistance for each of the six devices is much lower than the measured total resistance and hence is neglected.

The effective resistance of a network of randomly oriented, interconnected CNTs is highly dependent on the concentration of CNTs that make up the network. One can define two critical CNT concentrations, $x_{t}$ and $x_{s}$, that represent percolation threshold and percolation saturation, respectively. The percolation threshold is defined as the concentration of CNTs that characterizes the onset of the development of conducting paths throughout the CNT network. Before the CNT concentration reaches $x_{t}$, the CNTs are well separated, and the network is non-conducting. As the CNT concentration reaches and surpasses $x_{t}$, percolation paths build up progressively until the concentration approaches $x_{s}$, at which a very large number of CNTs participate in the percolation process. Any further increase in the CNT concentration will have minimum effect on network conductivity. So, in the range of concentrations between $x_{t}$ and $x_{s}$ (commonly referred to as percolation transition region), the effective conductivity of the CNT network increases sharply.

**Figure 5:**
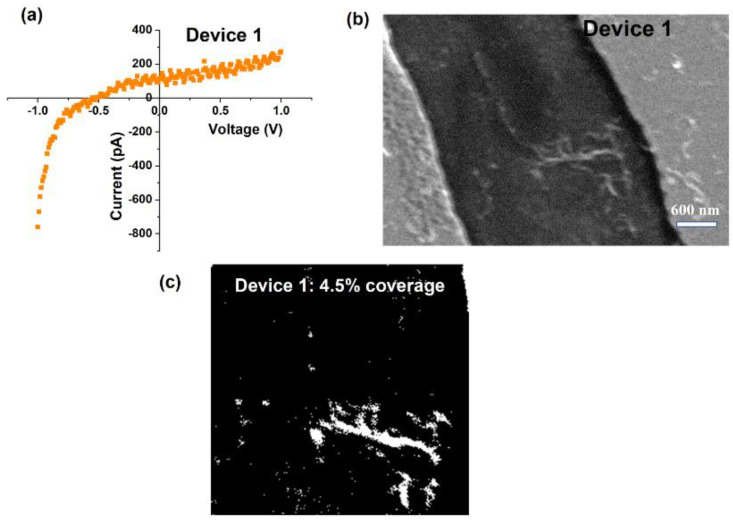
(**a**) Current-voltage behavior for device 1 showing negligible pA current, attributed to leakage across the surface, (**b**) SEM image of device 1 showing the two metal contacts and a very low concentration of MWCNTs in the 6 μm${}^{2}$ channel area, (**c**) Binary image of the SEM image shown in (**b**) with an estimated 4.5% of the channel area covered by MWCNTs.

We measured the effect of MWCNT density on sheet resistance of the CNT network and correlated this result to validate the 2D percolation behavior. The first set of measurements were performed on samples with very low MWCNT concentrations. Figure 5a shows the current-voltage plot for Device 1, which has almost no CNTs bridging the gap between the two metal contacts. This is evident in the SEM image shown in Figure 5b. In the absence of a percolating conducting path between the electrodes, the measured pA of current is most likely due to leakage currents. After converting the SEM image into a binary black/white image using Photoshop, the channel region was cropped out and analyzed with Image-J software (Figure 5c). Using the Count module in this software, the areal coverage of CNTs in the channel was determined as 4.5% of the total 6 μm${}^{2}$ channel area.

**Figure 6:**
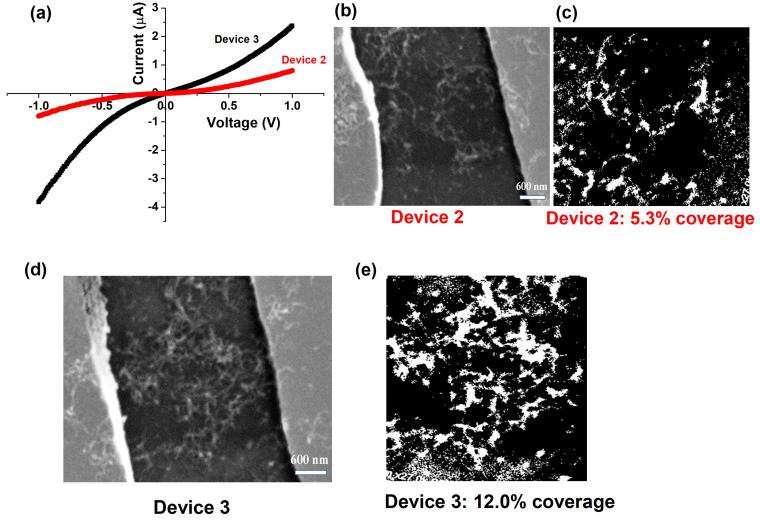
(**a**) Current-voltage characteristics for CNT devices 2 and 3 that have a significant number of interconnected MWCNTs in the network, (**b**,**c**) SEM image and corresponding binary image of device 2 showing few interconnected CNTs in the channel, corresponding to an areal coverage of 5.3%, (**d**,**e**) SEM image and corresponding binary image of device 3 showing a higher density of interconnected CNTs in the channel, corresponding to an areal coverage of 12.0%.

The second set of measurements were done on two devices containing a higher density of interconnected CNTs in the network. As the packing density increased, there was a significant increase in the measured current through the network (Figure 6).

As seen in the plot, at an areal coverage of 5.3%, device 2 is very resistive and has a resistance of 6.6 MΩ. In device 3, the CNT coverage increased to 12.0% and there is a significant increase in the current through the percolated network, with a measured resistance of 614 kΩ. It is also noted that devices 2 and 3 that had 5–10% coverage showed non-linear current–voltage behavior (Figure 6). As seen in this plot, the trend is linear at low bias and the network shows high resistance. This is because in the low bias regime the magnitude of the current follows Ohm’s law, where I ∝ V. The cross-over point in the current-voltage behavior occurs at about 0.2 V, and the most likely cause for this is the injection of electrons from the electrodes at high bias fields. Since the contact dimensions are much larger than that of the CNTs in the network, the behavior of the current variation with bias in this regime is expressed by Mott–Gurney law in which I ∝$V^{2}$.

**Figure 7:**
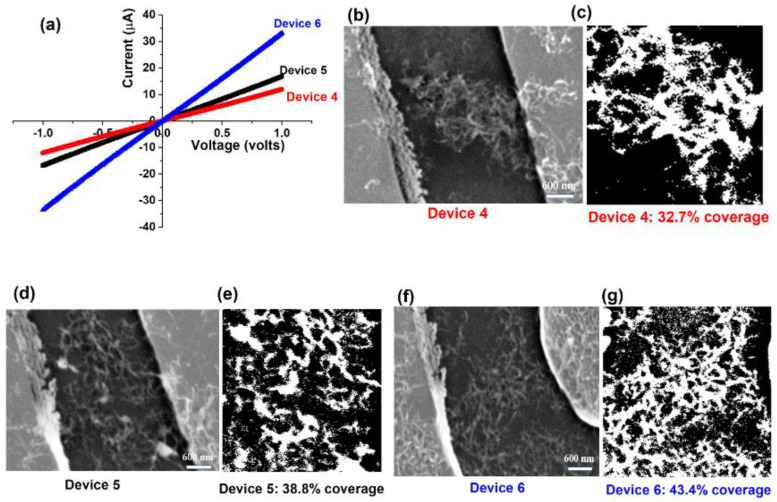
(**a**) Current-voltage characteristics for three CNT devices that have a significant number of interconnected MWCNTs in the network, (**b**,**c**) SEM image and corresponding binary image of device 4 showing interconnected CNTs in the channel, corresponding to an areal coverage of 32.7%, (**d**,**e**) SEM image and corresponding binary image of device 5 showing a higher density of interconnected CNTs in the channel, corresponding to an areal coverage of 30.8%, (**f**,**g**) SEM image and corresponding binary image of device 6 showing a very high density of interconnected CNTs in the channel, corresponding to an areal coverage of 43.4%.

In the third set of measurements, three devices containing a large number of interconnected MWCNTs in a dense network were studied. The current–voltage plot for these devices is shown in Figure 7.

Device 4 with a CNT areal coverage of 32.7% has a resistance of about 82.2 kΩ, while device 5 and 6 with coverage of 38.8% and 43.4% has resistance of 60.5 kΩ and 28.7 kΩ, respectively. It is also worth noting that CNT networks that had a high CNT concentration of CNTs in the channel showed a linear dependence of current on electrical bias (Figure 7).

### 3.3. Determination of Sheet Resistance and Analyzing Its Dependence on MWCNT Concentration

**Figure 8:**
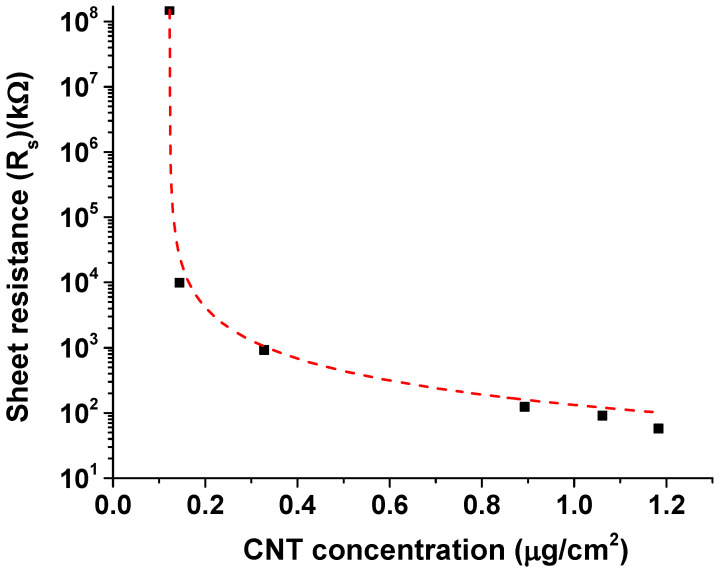
Variation in sheet resistance with CNT concentration. The percolation exponent is 1.42, which confirms transport by individual conducting paths in a two-dimensional network.

There are published works on percolation in transparent single-wall CNTs [33,34,35]. To compare our results with these published works, the resistance measured for each of the networks shown in Figure 5, Figure 6 and Figure 7 was converted to sheet resistance ($R_{s}$), which is defined as: $R_{s}$ = R(W/L), where R is the measured resistance and W and L are the width and length of the channel, respectively. The six devices that were characterized have different MWCNT concentration within the network and hence different percolation paths. In order to determine the percolation threshold value ($x_{t}$), it is essential to develop a methodology to assess the concentration of CNTs within the channel between the two electrical contacts. In previous works, for SWCNTs, this was done by correlating CNT concentration to optical absorption/transmission measurements [34,35]. In this work, we use a novel strategy of image processing to determine areal coverage by CNTs and use this result to determine surface concentration in units of mass per unit area. The volume density of CNTs purchased from NanoIntegris is 2.1 g/cm${}^{3}$. The CNTs in the network had an average outer diameter of about 15.0 nm, as determined by the SEM. Considering the network to be just a single layer of CNTs, the surface density of CNT was calculated to be about 3.0 μg/cm${}^{3}$. Considering that the channel over which the CNTs are distributed is about 6 μm${}^{2}$, it is possible to estimate the surface concentration of CNTs in the channel. The variation of sheet resistance $R_{s}$ with the MWCNT surface concentration measured in units of μg/cm${}^{2}$ is shown in Figure 8. Statistical percolation theory estimates the sheet resistance ($R_{s}$) of percolated thin films of one-dimensional nanomaterials to be described by percolation equation [33,34,35]:

(1) $$R_{s}=A{\left(x-x_{t}\right)}^{-\alpha }$$

where *A* is a scaling factor related to the intrinsic conductivity of CNT network in the high concentration limit, x is the MWCNT concentration, $x_{t}$ is the percolation threshold, and α is the percolation exponent related to the network dimensionality. For a two-dimensional network, the theoretical estimate for α is 1.33.

Following the fitting procedure discussed by Tenent et al. [35], our data in the range of 0.1 to 1.2 μg/cm${}^{2}$ were initially fit to a non-linear curve corresponding to the percolation equation to obtain a value of 110 Ω/square for the pre-factor A. Holding this value constant, the curve was fit to equation 1 to obtain the best fit, shown by the dashed red line in Figure 8. The percolation exponent α was determined to be 1.42 ± 0.07. This value is close to but slightly higher than the theoretically predicted value of 1.33 that is expected for electron transport by conducting pathways in a two-dimensional network [44]. There could be several reasons why the experimental and theoretical values of the exponent would differ. Errors in measurement, not enough data points around the critical point, as well as the possibility that the CNT film is not perfectly two-dimensional, are all factors that could account for the slightly elevated value of the exponent. The percolation threshold ($x_{t}$) as determined by the fit was 0.12 ± 4.5 ×$10^{-4}$μg/cm${}^{2}$. At this concentration, the resistance decreases several orders of magnitude as continuous electron paths form within the CNT network—the condition representing the onset of electrical conduction. For CNT concentrations *x* below the threshold value (device 1), there exist very few conduction pathways between the two contacts and, therefore, this yields a negligible off current (insulating state). As the MWCNT concentration in the network increases above the percolation threshold, several parallel pathways for electron transport open up and the network begins to conduct, eventually reaching a saturation level where the resistance stays almost constant (conducting state).

### 3.4. Variation of Sheet Resistance with CNT Network Complexity as Determined by MWCNT Concentration

In the preceding section, the CNT concentration is determined, based on estimates of areal coverage and surface density of CNTs in the network. Since it is not possible to quantify the number of CNTs in a network by counting them individually, a technique used in biological systems to study geometrical complexity by fractal analysis [45] is adopted for use in this work. The goal is to define a numerical index for the two-dimensional CNT network behavior, using a fractal dimension factor (D), that provides a statistical measure on how details in the network pattern change with the scale at which it is measured.

**Figure 9:**
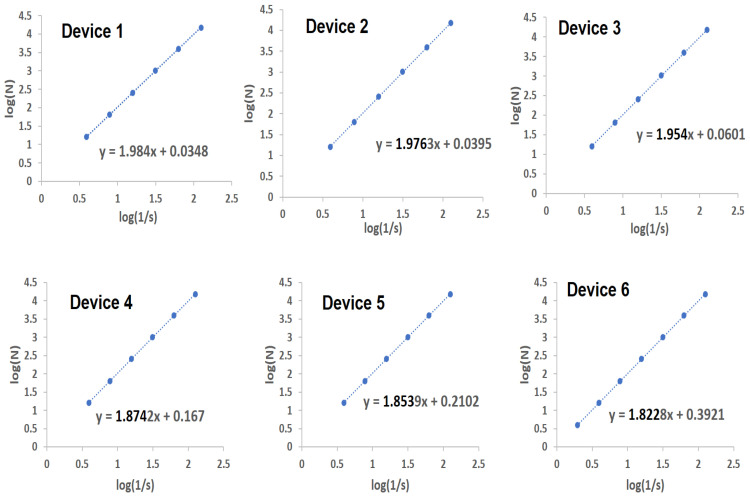
Determination of fractal dimension based on the contrast images for each of the six devices.

To facilitate this study, the binary black and white contrast images of the six devices (obtained using Image J) were used to determine the fractal dimensions of the CNT network. This was computed by Fractalyse software using a box counting algorithm. The fractal dimension (D) is determined by the equation [37,45]:

(2) $$D=\lim_{s\to 0}\frac{logN(s)}{log(1/s)}$$

where *s* is the length of the box which creates a mesh covering the CNT network, and *N*(*s*) is the minimal number of boxes which are required to cover the entire pattern. This method counts the number of boxes (*N*) of length s required to cover the object being measured. The variation between log(N) and log(1/s) is shown in Figure 9 for all six devices under study.

A distinctive characteristic of the six devices is the spatial complexity of the CNT network, where CNTs of different sizes are linked together. These networks are considered as fractal objects because of their irregular configuration, non-integer dimension and dependence on the scale of observation. As seen in the plot, the D value is different for each device, indicating its dependence on the irregular arrangement of CNTs in the network. Device 1, characterized by a very low density of CNTs in the network, has a D value of 1.984, while device 6 with a very high density of CNTs in network has a value of 1.822. The D-factor thus provides an estimate of the 2D geometrical complexity of the CNT network. As the value of D tends towards 2, its geometrical complexity is lower, because the network has a low density of CNTs. As the number of CNTs in the device is increased (more dense network), the D-factor decreases, since the complexity of the network is increased. The fractal dimension D is therefore inversely proportional to the areal coverage of the substrate by CNTs. This work is useful, since it provides a direct measurement of the areal coverage of a given surface by CNTs.

Fractal dimension D is therefore an important parameter that evaluates CNT network formation. In a recent report, Zhang et al. [46] used fractal dimension analysis to correlate the concentration of dispersed MWCNTs to D. They found that, in the lower concentration range from 0.01 to 10 wt%, D is proportional to the concentration of MWCNTs. On the other hand, at higher MWCNT concentrations of 10 to 15 wt%, D is inversely proportional to the concentration. In our work, the CNTs are dispersed on substrates and not bound within a matrix. This results in a more uniform dispersion of CNTs without the formation of agglomerates. As with the finding of Zhang et al. we observe an inverse relationship between D and CNT concentration, though our D values are slightly higher.

**Figure 10:**
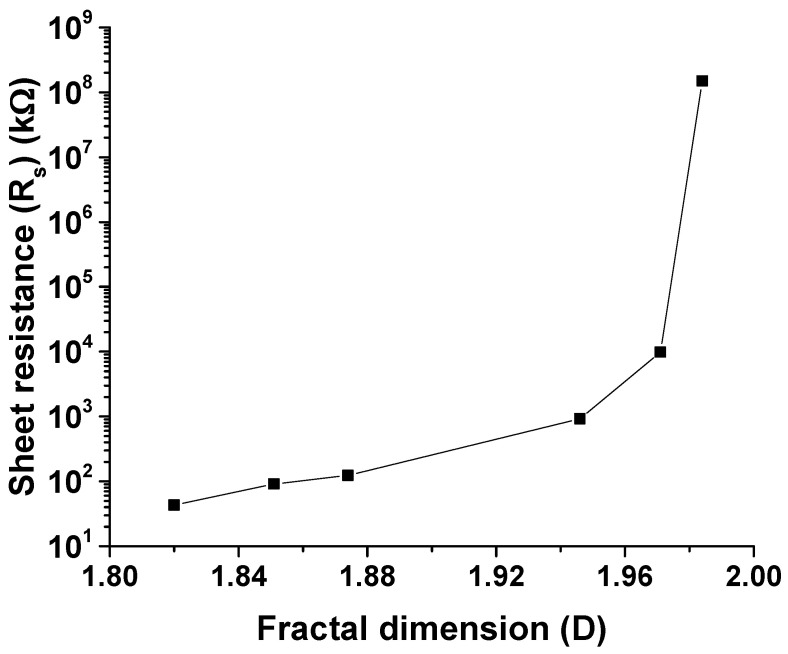
Variation in sheet resistance with fractal dimension of a given CNT network. The fractal dimension is inversely proportional to the MWCNT concentration in the network.

The relationship between network resistance ($R_{s}$) and the network complexity represented by the fractal dimension (D) is shown in Figure 10.

The plot validates that a network of CNTs that has reached or exceeded percolation saturation has a low “D” factor and a low sheet resistance, attributed to the fact that a more complex network of CNTs has several conduction pathways.

The experimentally determined value of percolation threshold ($x_{t}$), as 0.12 μg/cm${}^{2}$, is in good agreement with those reported for MWCNTs dispersed in thick polysulfone films [47]. Since our results on dispersed MWCNTs with no polymer matrix agree well with $x_{t}$ values reported for MWCNT composites [47], and also SWCNT composites [33,35], it is unclear what role the polymer and the type of CNT in the composite plays in the overall conduction process. It is possible that the polymer does not coat the individual CNTs, but rather coats the CNT network as a whole, facilitating more aggregation of CNTs in the network [48]. The scalable process reported in this work with regard to CNT dispersion and quantification are easily adaptable to producing large area films on any flexible or rigid patterned substrate, making the study well suited for many applications that require conducting CNT films.

## 4. Conclusions

We report on our findings of electrical resistance measurements on MWCNT networks. Using a meniscus-dragging deposition technique, we demonstrated the effective production of conducting CNT networks using a very small volume of the CNT suspension solution on Si/SiO${}_{2}$ and quartz substrates. Electron transport within the randomly interconnected MWCNT network was well described by the 2D-percolation theory. Considering that the MWCNTs are in intimate contact with each other, the measured resistance is attributed to contact between CNTs. Six devices with different areal coverage of CNTs covering a 6.0 μm${}^{2}$ channel area between two gold electrodes were studied. The sheet resistance of the devices was found to depend strongly on the MWCNT concentration. For networks that have an areal coverage below 4% (corresponding to a CNT concentration of less than 0.1 μg/cm${}^{2}$), the device is insulating. The resistance decreases by more than six orders of magnitude as the CNT concentration approaches 0.2 μg/cm${}^{2}$. This is attributed to an increase in the number of CNT–CNT junctions and the formation of several conducting pathways after CNT concentration exceeds the percolation threshold. Analyzing the experimental data in terms of the percolation theory yielded values for the percolation threshold $x_{t}$ and percolation exponent (α) as 0.12 μg/cm${}^{2}$ and 1.42, respectively. These results are quite similar to previously published results where CNTs are used as conductive fillers in a polymer matrix. A novel strategy to quantify CNT concentrations in a given network using image processing is presented along with the use of Fractalyse software to relate the percolation threshold to network complexity. Further investigation of electrical properties of the composite films is underway in our laboratory to understand the mechanism of electron conduction in such percolated networks.

## Abbreviations

The following abbreviations are used in this manuscript:

CNT	carbon nanotubes
SWCNT	single wall carbon nanotubes
MWCNT	multi-wall carbon nanotubes
MDD	meniscus dragging deposition
SEM	scanning electron microscope
SDS	sodium dodecyl-sulfate
Si/SiO${}_{2}$	silicon/silicon dioxide

## References

[1] Oberlin A., Endo M., Koyama T. (1976). Filamentous growth of carbon through benzene decomposition. J. Cryst. Growth.

[2] Iijima S. (1991). Helical microtubules of graphitic carbon. Nature.

[3] Shulaker M.M., Hills G., Patil N., Wei H., Chen H.-Y., Wong H.-S.P., Mitra S. (2013). Carbon nanotube computer. Nature.

[4] Alturaif H.A., ALOthman Z.A., Shapter J.G., Wabaidur S.M. (2014). Use of Carbon Nanotubes (CNTs) with Polymers in Solar Cells. Molecules.

[5] Ong P.-L., Euler W.B., Levitsky I.A. (2010). Hybrid Solar Cells Based on Single-Walled Carbon nanotubes/Si Heterojunctions. Nanotechnology.

[6] Pradhan B., Setyowati K., Liu H., Waldeck D.H., Chen J. (2008). Carbon Nanotube-Polymer Nanocomposite Infrared Sensor. Nano. Lett..

[7] Rao F., Liu X., Li T., Zhou T., Wang Y. (2009). The Synthesis and Fabrication of Horizontally Aligned Single-Walled Carbon Nanotubes Suspended Across Wide Trenches for Infrared Detecting Application. Nanotechnology.

[8] Itkis M.E., Borondics F., Yu A., Haddon C.J. (2006). Bolometric Infrared Photoresponse of Suspended Single-Walled Carbon Nanotube Films. Science.

[9] Sood A.K., Egerton E.J., Puri Y.R., Fernandes G., Kim J.H., Xu J., Goldsman N., Dhar N.K., Wijewarnasuriya P.S., Lineberry B.I. (2011). Carbon Nanotube based Microbolometer Development for IR Imager and Sensor Applications. Proc. Spie Infrared Sens. Devices Appl. Single Photon Imaging.

[10] Lu R., Li Z., Xu G., Wu J.Z. (2009). Suspending single-wall carbon nanotube thin film infrared bolometers on microchannels. Appl. Phys. Lett..

[11] Aliev A.E. (2008). Bolometric detector on the basis of single wall carbon nanotubes/polymercomposite. Infrared Phys. Technol..

[12] Glamazda A.Y., Karachevtsev V.A., Euler W.B., Levitsky I.A. (2012). Achieving High Mid-IR Bolometric Responsivity for Anisotropic Composite Materials from Carbon Nanotubes and Polymers. Adv. Funct. Mater..

[13] Fernandes G.E., Kim J.H., Sood A.K., Xu J. (2013). Giant temperature coefficient of resistance in carbon nanotube/phase-change polymer nanocomposites. Adv. Funct. Mater..

[14] Lu R., Shi J.J., Baca F.J., Wu J.Z. (2010). High performance multiwall carbon nanotube bolometers. J. Appl. Phys..

[15] Tarasov M., Svensson J., Kuzmin L., Campbell E.E.B. (2007). Carbon nanotube bolometers. Appl. Phys. Lett..

[16] Lu R., Xu G., Wu J.Z. (2008). Effects of thermal annealing on noise property and temperature coefficientof resistance of single-walled carbon nanotube films. Appl. Phys. Lett..

[17] Doherty E.M., De S., Lyons P.E., Shmeliov A., Nirmalraj P.N., Scardaci V., Joimel J., Blau W.J., John J., Boland J.J. (2009). The spatial uniformity and electromechanical stability of transparent, conductive films of single walled nanotubes. Carbon.

[18] Curran S.A., Talla J., Dias S., Zhang D., Carroll D., Birx D. (2009). Electrical transport measurements of highly conductive carbon nanotube/poly(bisphenol A carbonate) composite. J. Appl. Phys..

[19] Singh I., Bhatnagar P.K., Mathur P.C., Kaur I., Bharadwaj L.M., Pandey R. (2008). Optical and electrical characterization of conducting polymer-single walled carbon nanotube composite films. Carbon.

[20] Wang T., Lei C.-H., Dalton A.B., Creton C., Lin Y., Fernando K.A.S., Sun Y.-P., Manea M., Asua J.M., Keddie J.L. (2006). Waterborne, nanocomposite pressure sensitive adhesives with high tack energy, optical transparency, and electrical conductivity. Adv. Mater..

[21] Grossiord N., Kivit P.J.J., Loos J., Meuldijk J., Kyrylyuk A.A., van der Schoot P., Koning C.E. (2008). On the influence of the processing conditions on the performance of electrically conductive carbon nanotube/polymer nanocomposites. Polymer.

[22] Peng H., Sun X. (2009). Highly aligned carbon nanotube/polymer composites with much improved electrical conductivities. Chem. Phys. Lett..

[23] Ma P.C., Tang B.Z., Kim J.-K. (2008). Effect of CNT decoration with silver nanoparticles on electrical conductivity of CNT-polymer composites. Carbon.

[24] Sandler J.K.W., Kirk J.E., Kinloch I.A., Shaffer M.S.P., Windle A.H. (2003). Ultra-low electrical percolation threshold in carbon-nanotube-epoxy composites. Polymer.

[25] Li C.Y., Thostenson E.T., Chou T.W. (2008). Effect of nanotube waviness on the electrical conductivity of carbon naniotube based composites. Compos. Sci. Technol..

[26] Pan Y., Weng G.J., Meguid S.A., Bao W.S., Zhu Z.-H., Hamouda A.M.S. (2011). Percolation threshold and electrical conductivity of a two-phase composite containing randomly oriented ellipsoidal inclusions. J. Appl. Phys..

[27] Fu M., Yu Y., Xie J.J., Wang L.P., Fan M.Y., Jiang S.L., Zeng Y.K. (2009). Significant influence of film thickness on the percolation threshold of multiwall carbon nanotube/low density polyethylene composite films. Appl. Phys. Lett..

[28] Jiang M.J., Dang Z.M., Xu H.P. (2007). Giant dielectric constant and resistance-pressure sensitivity in carbon nanotubes/rubber nanocomposites with low percolation threshold. Appl. Phys. Lett..

[29] Hu N., Karube Y., Yan C., Masuda Z., Fukunaga H. (2008). Tunneling effect in a polymer/carbon nanotube nanocomposite strain sensor. Acta Mater..

[30] Zare Y., Rhee K.Y. (2020). Simulation of Percolation Threshold, Tunneling Distance, and Conductivity for Carbon Nanotube (CNT)-Reinforced Nanocomposites Assuming Effective CNT Concentration. Polymers.

[31] Gojny F.H., Wichmann M.H.G., Fiedler B., Kinloch I.A., Bauhofer W., Windle A.H., Schulte K. (2006). Evaluation and identification of electrical and thermal conduction mechanisms in carbon nanotube/epoxy composites. Polymer.

[32] Li C.Y., Thostenson E.T., Chou T.W. (2007). Dominant role of tunneling resistance in the electrical conductivity of carbon nanotube-based composites. Appl. Phys. Lett..

[33] Ko Y., Kim N.H., Lee N.R., Chang S.T. (2014). Meniscus-dragging deposition of single-walled carbon nanotubes for highly uniform, large-area, transparent conductors. Carbon.

[34] Hu L., Hecht D.S., Gru1ner G. (2004). Percolation in Transparent and Conducting Carbon Nanotube Networks. Nano Lett..

[35] Tenent R.C., Barnes T.M., Bergeson J.D., Ferguson A.J., To B., Gedvilas L.M., Heben M.J., Blackburn J.L. (2009). Ultrasmooth, Large-Area, High-Uniformity, Conductive Transparent Single-Walled-Carbon-Nanotube Films for Photovoltaics Produced by Ultrasonic Spraying. Adv. Mater..

[36] Bao W.S., Meguid S.A., Zhu Z.H., Weng G.J. (2012). Tunneling resistance and its effect on the electrical conductivity of carbon nanotube nanocomposites. J. Appl. Phys..

[37] Shao G., Xue X.X., Zhou X., Xu J., Jin Y., Qi S., Liu N., Duan H., Wang S., Li S. (2019). Shape-Engineered Synthesis of Atomically Thin 1T-SnS2 Catalyzed by Potassium Halides. ACS Nano.

[38] Schneider C.A., Rasband W.S., Eliceiri K.W. (2012). NIH Image to ImageJ: 25 years of image analysis. Nat. Meth..

[39] Abramoff M.D., Magalhaes P.J., Ram S.J. (2004). Image Processing with ImageJ. Biophot. Int..

[40] Rossi J.E., Soulea K.J., Cleveland E., Schmuckerc S.W., Cressc C.D., Coxa N.D., Merrilla A., Landi B.J. (2017). Removal of sodium dodecyl sulfate surfactant from aqueous dispersionsof single-wall carbon nanotubes. J. Colloid Interface Sci..

[41] Gao J., Wang W.Y., Cui L.J., Chen L.T., Hu X.Y., Li H., Geng H.Z. (2013). Effect of different concentrations of nitric acid on the conductivity of single-walled carbon nanotube transparent films. Adv. Mater. Res..

[42] Jaber-Ansari L., Iddir H., Curtiss L.A., Hersam M.C. (2014). Influence of electronic type purity on the lithiation of single-walled carbon nanotubes. ACS Nano.

[43] Salfi J., Philipose U., De Sousa C.F., Aouba S., Ruda H.E. (2006). Electrical properties of Ohmic contacts to ZnSe nanowires and their application to nanowire-based photodetection. Appl. Phys. Lett..

[44] Clerc J.P., Giraud G., Laugier J.M., Luck J.M. (1990). The electrical conductivity of binary disordered systems, percolation clusters, fractals and related models. Adv. Phys..

[45] Grizzi F., Russo C., Colombo P., Franceschini B., Frezza E.E., Cobos E., C-Internati M. (2005). Quantitative evaluation and modeling of two-dimensional neovascular network complexity: The surface fractal dimension. BMC Cancer.

[46] Zhang M., Zhang W., Jiang N., Futaba D.N., Xu M. (2019). A general strategy for optimizing composite properties by evaluationg the interfacial surface area of dispersed carbon nanotubes by fractal dimension. Carbon.

[47] Aguilar J.O., Bautista-Quijano J.R., Avilés F. (2010). Influence of carbon nanotube clustering on the electrical conductivity of polymer composite films. eXPRESS Polym. Lett..

[48] Ugur S., Yargi O., Pekcan O. (2010). Conductivity percolation of carbon nanotubes (CNT) in polystyrene (PS) latex film. Can. J. Chem..

